# Heavy Chalcogenide‐Based Ionic Liquids in Syntheses of Metal Chalcogenide Materials near Room Temperature

**DOI:** 10.1002/open.202000346

**Published:** 2021-02-10

**Authors:** Jannick Guschlbauer, Jörg Sundermeyer

**Affiliations:** ^1^ Fachbereich Chemie and Materials Science Center Philipps-Universität Hans-Meerwein-Str. 4 35032 Marburg Germany

**Keywords:** Ionic liquids, chalcogenido metalates, chalcogenide materials, photovoltaics, thermoelectrics

## Abstract

This minireview describes two strategically different and unexplored approaches to use ionic liquids (IL) containing weakly solvated and highly reactive chalcogenide anions [E‐SiMe_3_]^−^ and [E−H]^−^ of the heavy chalcogens (E=S, Se, Te) in materials synthesis near room temperature. The first strategy involves the synthesis of unprecedented trimethylsilyl chalcogenido metalates Cat^+^[M(E‐SiMe_3_)_n_]^−^ (Cat=organic IL cation) of main group and transition metals (M=Ga, In, Sn, Zn, Cu, Ag, Au). These fully characterized homoleptic metalates serve as thermally metastable precursors in low‐temperature syntheses of binary, ternary and even quaternary chalcogenide materials such as CIGS and CZTS relevant for semiconductor and photovoltaics (PV) applications. Furthermore, thermally and protolytically metastable coinage metalates Cat^+^[M(ESiMe_3_)_2_]^−^ (M=Cu, Ag, Au; E=S, Se) are accessible. Finally, the use of precursors BMPyr[E‐SiMe_3_] (E=Se,Te; BMPyr=1‐butyl‐1‐methylpyrrolidinium) as sources of activated selenium and tellurium in the synthesis of high‐grade thermoelectric nanoparticles Bi_2_Se_3_ and Bi_2_Te_3_ is shortly highlighted. The second synthesis strategy involves the metalation of ionic liquids Cat[S−H] and Cat[Se−H] by protolytically highly active metal alkyls or amides R_n_M. This rather general approach towards unknown chalcogenido metalates Cat_m_[R_n‐1_M(E)]_m_ (E=S, Se) will be demonstrated in a research paper following this short review head‐to‐tail.

## Graphical abstract

**Low temperature materials syntheses**: Ionic liquids with highly reactive anions [E‐SiMe_3_]^−^ and [E−H]^−^ (E=S, Se, Te) are valuable synthons for the synthesis of chalcogenido metalates Cat^+^[M(E‐SiMe_3_)_*n*_] and Cat_*n*_[R_*m*_M(E)]_*n*_ serving as thermally metastable precursors in binary and multinary metal chalcogenide materials preparation.

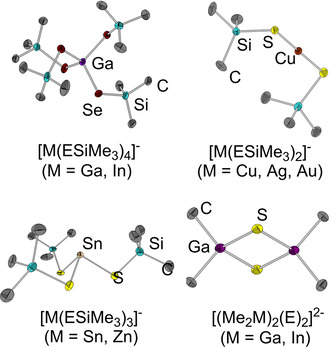

## 1 Introduction

For a very long period of time thiocyanate ILs were the only ionic liquids displaying a sulfur centered anion.^[1]^ In 2009, Earle, Seddon and coworkers presented ionic liquids with dithiocarbamate, xantogenate and organotrithiocarbonate anions, prepared via ion exchange of their sodium salts.^[2]^ Furthermore, quaternary ammonium hydrogen sulfide and polysulfide ILs have been synthesized by salt metathesis of Na_2_S hydrate in acetone / water with BMPyr[Cl] as ion exchange component.^[3]^ Due to these ion exchange synthesis methods all of these sulfur based redox electrolytes have not been obtained in any reported elemental or even electronic grade analytical purity. This metathesis method implies that water, hydroxide, chloride, sodium cations, and acetone condensation products are typically found as impurities, which cannot be accepted e. g. in electrochemical devices. Nevertheless such impure polysulfide electrolyte systems have been used as solvent for sulfur and as polysulfide IL redox mediators in quantum dot (CdS/TiO_2_) sensitized solar cells.^[3]^

Recently, we introduced a highly atom efficient synthesis of this class of ionic liquids and organic cation salts comprising chalcogenide based anions such as [SH]^−^, [SeH]^−^, [TeH]^−^,[^4^, ^5^, ^6^] [S‐SiMe_3_]^−^, [Se‐SiMe_3_]^−^ and [Te‐SiMe_3_]^−^,[^6^, ^7^] [S*t*Bu]^−^,^[8]^ and finally [S_x_]^2−^, [Se_x_]^2−^, [Te_x_]^2−^, and [Te_12_]^4−^.[^5^, ^6^, ^7^] The low temperature syntheses of these salts starts with methycarbonate ionic liquids Cat[OCO_2_Me], probably the cheapest ionic liquids on market. They are obtained via halide and metal free reaction of nucleophilic organic cation precursor molecules with dimethylcarbonate.^[9]^ This so‐called “green” waste‐free dimethylcarbonate route to ILs, the methylation reaction of tert.‐amines, ‐phosphines, N‐alkylimidazoles, N‐alkylpyrolidines by dimethylcarbonate in methanol at 120 °C under solvothermal conditions was further developed by us to an ionothermal synthesis of guanidinium methylcarbonates.^[10]^ The reaction of pentaalkyl guanidines and dimethylcarbonate can be conducted even without any added organic solvent.

**Scheme 1:**
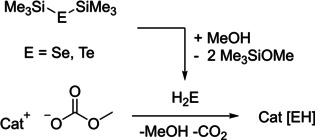
Representative hydrogenchalcogenide IL syntheses (E=S, Se, Te).[^4^, ^5^, ^6^]

**Scheme 2:**
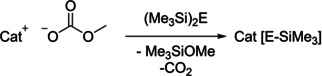
Representative trimethylsilyl‐chalcogenide IL syntheses (E=S, Se, Te).[^6^, ^7^]

It was discovered that these methylcarbonate ILs – depending on the solvent used – can act either as desilylating agent towards trimethylsilyl chalcogenides or as a base towards in situ protolytically formed EH_2_ (E=S, Se, Te). Typical protolysis reactions are presented in Scheme 1, desilylation reactions are presented in Scheme 2.

Both of these chalcogenide ILs classes Cat[EH] and Cat[E‐TMS] readily dissolve elemental chalcogens to form organic cation polychalcogenides such as single crystals of e. g. [DMPyr]_2_S_6_, [BMPyr]_2_Se_3_, and [DMPyr]_4_Te_12_.[^5^, ^6^, ^7^]

So far, chemists had only very few synthetic building blocks for such reactive chalcogenide anions at hand, that combine all favourable attributes in one reactive synthon: highest purity, perfect solubility in organic solvents, low melting points, and highest reactivity towards electrophiles and Lewis acids. The thermal decomposition pathways of these salts have been investigated by experiment and theoretical studies.^[4]^ They depend on the stability of the organic cation towards nucleophilic anion attack. Some of these salts are room temperature ILs, stable even beyond 180 °C, some of them decompose via cation dealkylation before they melt, some have a reversible melting point below 100 °C, some of them – the EMIm and BMIm[SH] and [SeH] salts (BMIm=1‐butyl‐3‐methylimidazolium) – have a surprisingly high vapor pressure and sublime quantitatively at very low temperature 100 °C/10^−2^ mbar.^[4]^ Desilylation of trimethylsilyl element compounds by methylcarbonate ILs could be extended to group 15 precursors E(SiMe_3_)_3_ (E=P, Sb). This allows an entry into unusual organic cation polypnictogenide clusters of the Zintl‐type such as [R_4_P]_3_[Sb_11_^3−^] and a very simple quantitative approach to Cat[PCO] ionic liquids.^[11]^

## 2 Syntheses of Metal Chalcogenide Materials Near Room Temperature

Due to the limited synthetic access to analytically pure heavy chalcogenide‐based ionic liquids, the research of using them in syntheses of metal chalcogenide materials near room temperature was essentially unexplored until recently. The following seven chapters review our strategies and results to overcome this knowledge gap.

### 2.1 Mixed Sulfido‐Selenido Mercurates via Ionothermal IL Flux Syntheses

Ionothermal synthesis is a term referring to the preparation or manipulation of solid‐state materials by the use of inorganic salt fluxes or ILs as solvent and/or reactive medium. Advantages like reusability of the solvent, ionic solvation conditions at the saturation limit and a comparably low energy demand in such atom‐economic syntheses amplify the potential of this method and explain the fast emerging of this field of research.[^12^, ^13^] During ionothermal metal chalcogenide syntheses the IL often exceeds its function as plain solvent by acting as e. g. ion source for salt metathesis reactions,^[14]^ alkylation agent for clusters,^[15]^ as activator for starting materials ^[16]^ or as source of elements of the target compound. For the latter purpose we proposed to utilize our chalcogenide based ILs as highly reactive chalcogen sources in low‐temperature metal chalcogenide material syntheses. In a collaboration with the Dehnen group, mixed sulfido‐selenido mercurates K_2_Hg_6_Se_7_, Na_2_Hg_3_S_2.51_Se_1.49_, K_2_Hg_3_S_1.03_Se_2.97_, and K_2_Hg_3_S_2.69_Se_1.31_ were synthesized by ionothermal chalcogen exchange starting with corresponding selenido mercurates dispersed in a hydrosulfide ionic liquid EMIm[SH] flux at 100–120 °C.^[17]^ In contrast to their lighter congeners, these band gap tuned title compounds could not be synthesized by inorganic polychalcogenide salt flux techniques previously. The applied method mimics polychalcogenide flux conditions, while operating at much lower temperatures below the decomposition temperature of the ionic liquid.

### 2.2 Targeting Precursors for 2D Metal Chalcogenide Materials

Besides coulombic interactions, hydrogen bonds and anion cation pi‐interactions greatly determine the thermal behaviour of ILs, e. g. the rather surprising sublimation and condensation of chalcogenide based ILs with dialkylimidazolium cation.^[4]^ In order to better understand the thermal stability of such ILs and their chemical and physical behaviour, anion‐cation interactions in the crystalline and liquid phase were experimentally investigated by XRD and NMR analyses and correlated with the number and strength of anion‐cation hydrogen bonds.^[18]^ With this basic insight ahead, we draw our attention towards coordination chemistry. Homoleptic neutral complexes [M(E−H)_n_] or [M(E‐SiMe_3_)_n_] were unknown. The same applied to homoleptic metalates Cat[M(E−H)_n_] and trimethylsilylchalcogenido metalates Cat[M(E‐SiMe_3_)_n_] (Cat=organic IL cation). The latter were identified as challenging synthetic targets ‐ and finally synthesized in the presence of our heavy chalcogenide ILs for metals M=Ga, In, Sn. They were spectroscopically, crystallographically and thermoanalytically characterized and used as binary building blocks in liquid phase low‐temperature syntheses of binary and multinary metal chalcogenide materials. 2D metal sulfides and selenides of tin and gallium in particular are attractive targets: 2D‐SnS_2_ is a visible‐light photocatalyst, earth abundant, cheap and environmentally benign. 2D‐SnS_2_ can undergo exfoliation similar to 2D‐MoS_2_. An electron affinity of 4.16 eV, an ionisation potential of 6.44 eV and a work function of 4.81 eV make this material an ideal candidate for photocatalytic water splitting.^[19]^ 2D‐SnS is a brown‐black layered material, with can be exfoliated from single crystals. It shows a high optical absorption coefficient, p‐type conductivity, and a direct band gap of 1.3–1.4 eV. It is cheap, earth abundant and nontoxic, properties that qualify it as efficient PV absorber layer for thin‐film PV modules of the coming generations replacing CdTe and CIGS^[20]^ and 2D‐GaSe is a III–VI semiconductor material with an indirect band gap of 2.1 eV. It is photoconducting and reveals properties applied in non‐linear optics for frequency doubling.^[21]^ Other approaches to prepare these target materials usually follow thermolysis protocols of precursors stabilised by auxiliary ligands. Their presence might or might not be beneficial for the defined growth of nanoparticles.^[22]^

### 2.3 Approaching PV Materials Cu(In_*x*_Ga_1−*x*_)(S_*y*_Se_1−*y*_)_2_ (CIGS)

CIGS materials are among the best performing PV absorber materials known up to date. The common way to prepare solar panels of these materials involve either ecologically unfavourable sulfuration or selenation steps with large excesses of highly toxic H_2_E gas or expensive equipment for high‐temperature gas phase treatment by E_x_ radicals.^[23]^ Although solution‐based^[24]^ and single‐source strategies are known^[25]^ there is still a considerable demand for cheap, solution‐based syntheses of CIGS particles of reasonable performance.

By targeting this class of copper‐indium‐gallium‐sulfides and selenides CIGS, our focus was the synthesis and full characterization of so far unknown tetrakis(trimethylsilylsulfido) and ‐(trimethylsilylselenido) gallates and indates in form of their organic cation salts Cat^+^[M(ESiMe_3_)_4_]^−^ (M=Ga, In; E=S, Se; Cat^+^=DMPyr^+^, Ph_4_P^+^, (dppe)_2_Cu^+^, and (dmpe)_2_Cu^+^, dppe=diphenylphosphinoethane, dmpe=dimethylphosphinoethane).^[26]^ There is a small corridor with respect to the reaction conditions allowing the isolation and full characterization of these thermally metastable molecular precursors in the presence of trimethylsilyl‐chalcogenide ILs and MCl_3_. Only small deviations from the published protocol lead to premature precipitation of binary chalcogenides M_2_E_3_ and ME at room temperature instead of the isolation of soluble molecular metalate title complexes. These thermally metastable silylchalcogenido metalates can act as modular element precursors for an ionic‐liquid‐ or organic‐solution‐based low‐temperature synthesis of multinary metal chalcogenide materials such as the CIGS PV materials Cu(In_*x*_Ga_1−*x*_)(S_*y*_Se_1−*y*_)_2_. We developed a low‐temperature approach of using a solution (or ink in a proposed ink jet printed electronics process) of binary soluble precursors Cat^+^[M(ESiMe_3_)_4_]^−^ as source for the elements In/Ga and S/Se and a solution (or film of ink) of Cu^+^ ions in form of their tetramethylthiourea (tmtu) complexes [Cu(tmtu)_3_]PF_6_. After removing by‐products Cat[PF_6_] and tmtu, thermal annealing of the microcrystalline precipitate is leading to phase pure microcrystalline CIGS detected via PXRD.^[26]^ This method has a higher potential to be further developed into a larger scale printed CIGS process for PV modules than the single‐source precursor approach demonstrated as well: As Cu(+1) tends to show a higher affinity towards sulfur and selenium anions than Ga(3+) and In(3+), copper has to be masked by diphosphine ligands dppe or dmpe in order to isolate single source precursors with cations such as [(dppe)_2_Cu]^+^ or [(dmpe)_2_Cu]^+^[M(ESiMe_3_)_4_]^−^ (M=Ga, In; E=S, Se). Due to their high molecular mass, and higher load of waste components and ligand costs, this single‐source CIGS precursor approach is probably only of academic interest.

### 2.4 Approaching PV Materials Cu_2_ZnSn(S_x_Se_1−x_)_4_ (CZTS)

Another class of PV absorber materials incorporating more abundant, non‐toxic metals is represented by the class of copper‐zinc‐tin‐sulfides and selenides CZTS.^[27]^ In order to investigate potential approaches via molecular precursor complexes possibly forming in our ILs, we targeted the isolation of unprecedented metastable zincate and stannanide – or stannate(II) – precursors Cat^+^[M(ESiMe_3_)_3_]^−^ (M=Zn, Sn ; E=S, Se),^[28]^ thereby providing a possible alternative to established solution‐based processes for the preparation of Cu_2_ZnSnS_4_ relying on e. g. dithiocarbamate intermediates.^[29]^

Trimethylsilylsulfido‐ and ‐selenido zincates [Zn(ESiMe_3_)_3_]^−^ (E=S, Se) and corresponding stannanides [Sn(ESiMe_3_)_3_]^−^ (E=S, S) were isolated and fully characterized as their organic cation salts with Cat^+^=PPh_4_^+^ or Ph_3_PNPPh_3_^+^ (PPN). The smaller the organic cation, the more thermally labile is this class of compounds. Silylsulfido zincate Ph_4_P[Zn(SSiMe_3_)_3_] reveals a trigonal planar configuration in the XRD analysis, silylselenido zincate [(Me_3_SiSe)_2_Zn(μ‐SeSiMe_3_)]_2_^2−^ a tetrahedral configuration at zinc by forming a dianionic dinuclear complex, finally, Ph_4_P[Sn(SSiMe_3_)_3_] is coordinated in a trigonal pyramidal (pseudotetrahedral) fashion, as expected for a main group element. The thermal decomposition of these precursor compounds has been investigated by TGA measurements under inert gas. The TGA residues have been identified as corresponding binary chalcogenides ZnS, ZnSe, SnS and SnSe by PXRD. While these metalates, due to their anionic nature and large organic cation, are thermally rather stable, neutral tin(IV) derivatives [Sn(ESiMe_3_)_4_] (E=S, Se) are metastable even at room temperature. Nevertheless, they can be synthesized, NMR spectroscopically fully characterized and used as sources to precipitate SnS_2_ and SnSe_2_ from ILs or organic solution containing traces of water or MeOH. However, thermal decay of neutral stannanes [Sn(ESiMe_3_)_4_] above 0 °C does not allow storing them as inks for extended periods of time. Therefore, a different solution‐based approach towards CZTS particles starting from tin(II) derivative PPN[Sn(SSiMe_3_)_3_], its oxidation by elemental sulfur, subsequent addition of 1 eq. zincate PPN[Zn(SSiMe_3_)_3_] and 2 eq. of copper source [Cu(tmtu)_3_]PF_6_ in MeCN was investigated. The non‐crystalline black precipitate from this three‐component organic salt mix gave, after washing and annealing steps, microcrystalline quaternary chalcogenide material CZTS.^[18]^ Although further optimization is needed in order to get absolutely phase pure CZTS with ILs of smaller and cheaper cations, this study might be seen as fundamental investigation of an optional sol‐gel‐type condensation approach towards such multinary heavy chalcogenide materials. The challenge is to slow down the thermodynamically favoured precipitation of phase separated binary chalcogenides by organic IL cations. There are plenty of options by modifying the organic cations and solvents without changing the novel anionic Zn/Sn/S/Se sources described. Our results demonstrate, that CZTS particles can be prepared by co‐precipitation of non‐crystalline nanoparticles from organic solvents at very low temperatures such as 0 °C. Thus, metastable zincates and stannates(II) and stannanes [Sn(E‐SiMe_3_)_4_] (E=S, Se) can be applied in the precipitation of nano‐ and mircocrystalline SnE_2_ and SnE and ZnE precipitates as dispersions in organic solvents and ILs. The potential of using them as inks in printed electronics should be further investigated.

### 2.5 Metastable Coinage Metalates Cat^+^[M(ESiMe_3_)_2_]^−^ (M=Cu, Ag, Au; E=S, Se)

In the latest report with respect to the title story we describe the syntheses and crystallographically determined molecular structures of unprecedented homoleptic trimethylsilylsulfido and trimethylsilylselenido cuprates, argentates and aurates Cat^+^[M(SSiMe_3_)_2_]^−^ and Cat^+^[M(SeSiMe_3_)_2_]^−^ (M=Cu, Ag, Au) comprising stabilizing Ph_4_P^+^ or PPN^+^ cations.^[30]^ Much to our surprise these homoleptic coinage metalate anions are stable enough to be isolated even in the absence of any other strongly metal binding and stabilising ligands such as phosphines or N‐heterocyclic carbenes. The complete series of metalates with all six element combinations has been characterized by XRD analysis. The coinage metal atoms are coordinated by two trimethylsilylchalcogenido ligands in a linear fashion. The silyl moieties of all presented anions show an unexpected gauche conformation of the silyl substituents with respect to the central axis Si‐[E−M‐E]‐Si in the solid state. The energetic preference for the gauche conformation is confirmed by quantum chemical calculations and amounts to about 2–6 kJ/mol, thus revealing a rather shallow potential mainly depending on electronic effects of the metal. Furthermore, 2D HMQC methods were applied to detect the otherwise non‐observable NMR shifts of the ^29^Si‐ and ^77^Se‐nuclei of the silylselenido compounds. Preliminary investigations reveal, that these thermally and protolytically labile chalcogenido metalates are valuable precursors for the precipitation of binary coinage metal chalcogenide nanoparticles from organic solution or ILs and for coinage metal cluster syntheses.^[30]^

### 2.6 Thermoelectric Materials Bi_2_E_3_ via Activated Chalcogen Sources BMPyr[E‐SiMe_3_] (E=Se,Te)

In a collaboration with the groups of G. Schierning (Dresden) and S. Schulz (Duisburg‐Essen), our ionic precursors BMPyr[E‐SiMe_3_] (E=Se,Te) demonstrated their synthetic value as activated and highly reactive sources of selenium and tellurium. They were used in low‐temperature syntheses of phase‐pure tetradymite‐type crystalline Bi_2_Se_3_ and Bi_2_Te_3_ nanoparticles via their reactions with ionic precursor [BMIm]_3_[Bi_3_I_12_] (BMIm=1‐butyl‐3‐methylimidazolium).^[31]^ Notably, the surfaces of the Bi_2_Se_3_ and Bi_2_Te_3_ nanoparticles were free of any metal oxide species or contaminations by elemental tellurium or oleylamine additive typically observed in previous studies using Se(SiEt_3_)_2_ and Te(SiEt_3_)_2_ as selenium and tellurium sources.^[32]^ The nanoparticle surfaces of BMPyr[E‐SiMe_3_] (E=Se,Te) derived material reveal only traces of organic contamination from BMIm[I] solvent residues. Thus, these nanomaterials show high Seebeck coefficients of −124 μV K^−1^ (Bi_2_Se_3_) and −155 μV K^−1^ (Bi_2_Te_3_) and feature high electrical conductivities (328 and 946 S cm^−1^, respectively) at the highest tested temperature (240 °C). The corresponding thermal conductivities (0.8 and 2.3 W m^−1^ K^−1^, respectively, at 30 °C) are comparable to those of single crystals.

### 2.7 Metalation of Hydrochalcogenide Ionic Liquids Cat[S‐H] and Cat[Se‐H]

**Scheme 3:**
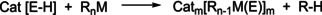
Chalcogenido metalate complex synthesis strategy via metalation of ionic liquids

This strategy involves reactions of protolytically highly active metal alkyls or amides R_n_M as metalating agents. If their functionality R is sufficiently basic to deprotonate hydrosulfide or hydroselenide anions pre‐coordinated at M, the result of this reaction would lead to a largely unexplored class of multinuclear (m) chalcogenido metalates Cat_m_[R_n‐1_M(E)]_m_ (E=S, Se). Scheme 3 represents this transformation in a general way for one group R^‐^ being substituted by one group E^2‐^. Place Scheme 3 here

The question arises, how much negative charge corresponding with m might possibly be accumulated in such condensation reactions towards multinuclear metal chalcogenide anions. This question and synthesis strategy is addressed in our report investigating the metalation of Cat[EH] (E=S, Se) with Me_3_Ga and Me_3_In. The primary condensation products turned out to be dianionic four‐membered rings Cat_2_[Me_2_M(μ_2_‐E)]_2_ (M=Ga, In, E=S, Se). Their ring cleavage, expansion, and condensation reactions are reported.^[33]^

## 3 Summary and Outlooks

During this scientific excursion into metal sulfide, selenide and telluride chemistry we learned to use our very pure and crystalline organic cation (Cat^+^) salts containing anions [SH]^−^, [SeH]^−^, [TeH]^−^, [S‐SiMe_3_]^−^, [Se‐SiMe_3_]^−^ and [Te‐SiMe_3_]^−^ and related ionic liquids as highly reactive, weakly solvated sources of the heavier chalcogen anions in the synthesis of binary and multinary metal chalcogenide materials and, in particular, their thermally metastable molecular precursors: A new class of unprecedented trimethylsilylsulfido‐ and ‐selenido metalates Cat^+^[M(E‐SiMe_3_)_n_]^−^ of main group and transition metals (M=Ga, In, Sn, Zn, Cu, Ag, Au) was synthesized, structurally and thermoanalytically characterised und used in the low temperature synthesis of nanocrystalline metal chalcogenides. In the synthesis of Bi_2_Se_3_ and Bi_2_Te_3_ nanoparticles, plausible intermediate bismuthates involving [Se‐SiMe_3_] and [Te‐SiMe_3_] ligands were not isolated. Instead, a very pure form of these high‐grade thermoelectric materials was synthesized and characterized by our collaborators. While precipitation of such binary, ternary, even quaternary metal chalcogenides can easily be accomplished with described precursors at room temperature from organic solution, in particular if traces of protic alcohols or water are added, it should be noted, that an annealing process at slightly higher temperatures (300–400 °C under inert gas) is needed in order to get microcrystalline, PXRD refractive and analytically pure metal chalcogenide phases in most of the cases. Our TGA/DSC/MS investigations on the thermal decomposition of quaternary ammonium and imidazolium chalcogenide salts presented in the introduction of this review clearly demonstrate, that such quaternary alkyl‐onium other than phenyl‐phosphonium salts fully degrade in one step with sharp offset temperatures below 230 °C to only volatile products without leaving any organic residual impurities at 1 bar. This observation might be taken as an advantage and vision to introduce these highly reactive sulfide, selenide and telluride sources into a solution‐based, print‐technology‐based production line of CIGS or CZTS photovoltaic modules. The advantage could be, that only slightly over‐stoichiometric amounts of reactive chalcogen sources would have to be applied without having to use and dispose an extremely large excess of toxic H_2_S, H_2_Se or E_x_ radicals generated at very high temperatures in the final module production process, which is the state of the art currently.

## Conflict of interest

The authors declare no conflict of interest.

## Supporting information

As a service to our authors and readers, this journal provides supporting information supplied by the authors. Such materials are peer reviewed and may be re‐organized for online delivery, but are not copy‐edited or typeset. Technical support issues arising from supporting information (other than missing files) should be addressed to the authors.

Supplementary

## References

[1] P. Wasserscheid , T. Welton , Ionic Liquids in Synthesis, 2 ^nd^ ed., vol. 1+2, Wiley-VCH, Weinheim 2008.

[2] E. Boros , M. J. Earle , M. A. Gilea , A. Metlen , A.-V. Mudring , F. Rieger , A. J. Robertson , K. R. Seddon , A. A. Tomaszowska , L. Trusov , J. S. Vyle , Chem. Commun. 2010, 46, 716–718. 10.1039/b910469k 20087497

[3] V. Jovanovski , V. Gonzalez-Pedro , S. Gimenez , E. Azaceta , G. Cabanero , H. Grande , R. Tena-Zaera , I. Mora-Sero , J. Bisquert , J. Am. Chem. Soc. 2011, 133, 20156–20159. 22107441 10.1021/ja2096865

[4] L. H. Finger , F. Wohde , E. I. Grigoryev , A.-K. Hansmann , R. Berger , B. Roling , J. Sundermeyer , Chem. Commun. 2015, 51, 16169–16172. 10.1039/c5cc06224a 26377144

[5] L. H. Finger , J. Sundermeyer , Chem. Eur. J. 2016, 12, 4218–4230. 10.1002/chem.201504577 26879604

[6] L. H. Finger, J. Sundermeyer (Univ. Marburg), WO2015078774 (A1); EP2876081 (A1).

[7] L. H. Finger , B. Scheibe , J. Sundermeyer , Inorg. Chem. 2015, 54, 9568–9575. 26371537 10.1021/acs.inorgchem.5b01665

[8] L. H. Finger , J. Guschlbauer , K. Harms , J. Sundermeyer , Chem. Eur. J. 2016, 22, 16292–16303. 27717038 10.1002/chem.201602973

[9] Representative examples of the dimethyl carbonate route for IL synthesis:

[9a] B. Albert , M. Jansen , Z. Anorg. Allg. Chem. 1995, 621, 1735–1740;

[9b] Z. Zheng , T. Wu , X. Zhou , Chem. Commun. 2006, 1864–1865; 10.1039/b601343k 16622510

[9c] R. Kalb , PCT Int. Appl., 2008/052 860, **2008**;

[9d] M. Fabris , V. Lucchini , M. Noè , A. Perosa , M. Selva , Chem. Eur. J. 2009, 15, 12273–12282; 19810060 10.1002/chem.200901891

[9e] M. Smiglak , C. C. Hines , R. D. Rogers , Green Chem. 2010, 12, 491–501.

[10a] B. Oelkers , J. Sundermeyer , Green Chem. 2011, 13, 608–618;

[10b] J. Sundermeyer, B. Oelkers (Univ. Marburg), WO2011095428 (A1), EP2354121 (A1).

[11] M. Jost , L. H. Finger , J. Sundermeyer , C. von Hänisch , Chem. Commun. 2016, 52, 11646–11648. 10.1039/c6cc06620h 27602991

[12] E. R. Cooper , C. D. Andrews , P. S. Wheatley , P. B. Webb , P. Wormald , R. E. Morris , Nature 2004, 430, 1012–1016. 15329717 10.1038/nature02860

[13a] S. Santner , J. Heine , S. Dehnen , Angew. Chem. Int. Ed. 2016, 55, 876–893; 10.1002/anie.201507736 26661858

[13b] M. F. Groh , U. Müller , E. Ahmed , A. Rothenberger , M. Ruck , Z. Naturforsch. 2013, 68b, 1108 – 1122.

[14] G. Thiele , S. Santner , C. Donsbach , M. Assmann , M. Müller , S. Dehnen , Z. Kristallogr. 2014, 0, 489–495.

[15] B. Peters , S. Santner , C. Donsbach , P. Vöpel , B. Smarsly , S. Dehnen , Chem. Sci. 2019, 10, 5211–5217. 31191876 10.1039/c9sc01358j PMC6540918

[16] T. Zhang , K. Schwedtmann , J. J. Weigand , T. Doert , M. Ruck , Chem. Commun. 2017, 53, 7588–7591. 10.1039/c7cc03564k 28638904

[17] C. Donsbach , G. Thiele , L. H. Finger , J. Sundermeyer , S. Dehnen , Inorg. Chem. 2016, 55, 6725–6730. 27299466 10.1021/acs.inorgchem.6b00974

[18] J. Guschlbauer , T. Vollgraff , J. Sundermeyer , Dalton Trans. 2019, 48, 10971–10978. 31210231 10.1039/c9dt01586h

[19] L. A. Burton , T. J. Whittles , D. Hesp , W. M. Linhart , J. M. Skelton , B. Hou , R. F. Webster , G. O′Dowd , C. Reece , D. Cherns , D. J. Fermin , T. D. Veal , V. R. Dhanak , A. Walsh , J. Mater. Chem. A 2016, 4, 1312–1318.

[20] J. A. Andrade-Arvizu , M. Courel-Piedrahita , O. Vigil-Galán , J. Mater. Sci. Mater. Electron. 2015, 26, 4541–4556.

[21] R. H. Bube , E. L. Lind , Phys. Rev. 1959, 115, 1159–1164.

[22a] K. Klauke , B. Hahn , K. Schütte , J. Barthel , C. Janiak , Nano-Structures & Nano-Objects 2015, 1, 24–31; Nano-Objects **2015**, 1, 24–31;

[22b] S. Heimann , W. Assenmacher , O. Prymak , S. Schulz , Eur. J. Inorg. Chem. 2015, 14, 2407–2415.

[23] J. Ramanujam , U. P. Singh , Energy Environ. Sci. 2017, 10, 1306–1319.

[24] T. K. Todorov , O. Gunawan , T. Gokmen , D. B. Mitzi , Prog. Photovoltaics 2013, 21, 82—87.

[25] W. Hirpo , S. Dhingra , A. C. Sutorik , M. G. Kanatzidis , J. Am. Chem. Soc. 1993, 115, 4, 1597–1599.

[26] J. Guschlbauer , T. Vollgraff , J. Sundermeyer , Inorg. Chem. 2019, 58, 15385–15392. 31687815 10.1021/acs.inorgchem.9b02453

[27] X. Liu , Y. Feng , H. Cui , F. Liu , X. Hao , G. Conibeer , D. B. Mitzi , M. Green , Prog. Photovolt.: Res. Appl. 2016, 24, 879–898.

[28] J. Guschlbauer , T. Vollgraff , J. Sundermeyer , Dalton Trans. 2020, 49, 2517–2526. 32022066 10.1039/c9dt04144c

[29] K. Liu , B. Yao , Y. Li , Z. Ding , H. Sun , Y. Jiang , G. Wang , D. Pan , J. Mater. Chem. C 2017, 5, 3035–3041.

[30] J. Guschlbauer, T. Vollgraff, X. Xie, F. Weigend, J. Sundermeyer, *Inorg. Chem* **2020**, *59*, 17565–17572**in print**, DOI: /10.1021/acs.inorgchem.0c02808. 10.1021/acs.inorgchem.0c02808 33197182

[31] M. Loor , S. Salloum , P. Kawulok , S. Izadi , G. Bendt , J. Guschlbauer , J. Sundermeyer , N. Pérez , K. Nielsch , G. Schierning , S. Schulz , Inorg. Chem. 2020, 59, 3428–3436. 31967797 10.1021/acs.inorgchem.9b03060

[32a] M. Loor , G. Bendt , U. Hagemann , C. Wölper , W. Assenmacher , S. Schulz , Dalton Trans. 2016, 45, 15326–15335; 27722333 10.1039/c6dt02361d

[32b] M. Loor , G. Bendt , J. Schaumann , U. Hagemann , M. Heidelmann , C. Wölper , S. Schulz , Z. Anorg. Allg. Chem. 2017, 643, 60–68.

[33a] J. Guschlbauer, Dissertation Marburg **2019**;

[33b] J. Guschlbauer , T. Vollgraff , L. H. Finger , K. Harms , J. Sundermeyer , ChemistryOpen 2021 accepted, following paper. 10.1002/open.202000347 PMC7874246 33565735

