# Supplementary material for: Heavy Chalcogenide‐Based Ionic Liquids in Syntheses of Metal Chalcogenide Materials near Room Temperature
Source: ChemistryOpen. 2021 Feb 10;10(2):92–6. doi: 10.1002/open.202000346 (PMC7874248; doi:10.1002/open.202000346)
Supplement: Supplementary file 1 — Supplementary [file OPEN-10-92-s001.pdf]

Author Contributions

J.S. Conceptualization:Equal; Project administration:Lead; Supervision:Lead; Validation:Lead; Writing – original draft:Equal; Writing – review & editing:Lead
J.G. Conceptualization:Equal; Formal analysis:Lead; Methodology:Equal; Visualization:Equal
